# Dose-Dependent Bioavailability and Tissue Distribution of the ATR Inhibitor AZD6738 (ceralasertib) in Mice

**DOI:** 10.1007/s00280-021-04388-x

**Published:** 2022-01-23

**Authors:** Brian F. Kiesel, Jianxia Guo, Robert. A Parise, Raman Venkataramanan, David A. Clump, Christopher J. Bakkenist, Jan H. Beumer

**Affiliations:** 1 Cancer Therapeutics Drug Discovery Program, University of Pittsburgh Cancer Institute, Pittsburgh, PA; 2 Department of Pharmaceutical Sciences, School of Pharmacy, University of Pittsburgh, Pittsburgh, PA; 3 Department of Pathology, School of Medicine, University of Pittsburgh, Pittsburgh, PA; 4 Department of Radiation Oncology, School of Medicine, University of Pittsburgh, Pittsburgh, Pennsylvania, PA; 5 Department of Pharmacology and Chemical Biology, School of Medicine, University of Pittsburgh, Pittsburgh, Pennsylvania, PA; 6 Division of Hematology-Oncology, Department of Medicine, University of Pittsburgh School of Medicine, Pittsburgh, PA

**Keywords:** Pharmacokinetics, small molecule inhibitor of ATR, AZD6738, tissue distribution, LC/MS

## Abstract

**Purpose:**

Ataxia telangiectasia and Rad3-related (ATR) initiates and regulates cellular responses to DNA damage, such as those caused by cancer treatments. Several ATR inhibitors (ATRi) are in clinical development including AZD6738. Therapeutic indices among ATRi may differ as a result of varying potencies and concentrations at both tumor and off-target sites. Additionally, AZD6738 contributes to anti-tumor immune responses necessitating evaluation of exposure at immunological sites.

**Methods:**

Using mouse models and a highly sensitive LC-MS/MS assay, the pharmacokinetics of AZD6738 were studied, including dose linearity, bioavailability, metabolism, and tissue distribution in tumor-bearing mice.

**Results:**

Initial studies identified dose-dependent bioavailability, with greater than proportional increases in exposure as dose increased resulting in a ~2-fold increase in bioavailability between the lowest and highest investigated doses. These behaviors were successfully captured with a compartmental PK model. Analysis of metabolite PK revealed decreasing metabolic ratios with increasing dose, indicative of saturable first-pass metabolism. Further analysis revealed that intestinal and gut metabolism contribute to metabolism and these saturable mechanisms. Studies of tumor and tissue distribution found rapid and extensive drug distribution to most tissues except brain and spinal cord.

**Conclusion:**

The complex non-linear behavior of AZD6738 PK in mice was due to pre-systemic saturation and which appears to be recapitulated clinically at low doses. PK reported here will allow future correlation of tissue related toxicities with drug exposure as well as exposure with immunological responses. These results can also be compared with those from similar studies of other ATRi to contrast drug exposure with responses.

## 1 INTRODUCTION

Cancer treatment employs chemotherapies and ionizing radiation (IR) that cause DNA damage and ultimately cell death in both malignant and healthy tissue. Highly coordinated repair processes, collectively known as the DNA damage response (DDR), limit tissue damage. An apical component of the DDR is the serine/threonine kinase Ataxia Telangiectasia and Rad3-related (ATR) which is activated and initiates a signaling cascade at damaged replication forks [1]. ATR is also activated at other structures containing extended regions of single-strand DNA such as those formed during the repair of DNA double-strand breaks (DSBs) by homologous recombination [2]. Central to the signaling cascade initiated by ATR is a second serine/threonine kinase CHK1 which is phosphorylated and activated by ATR, thereby extending the response [1,3]. Cancer cells that have acquired defects in certain aspects of the DDR, such as p53-dependent cell cycle arrest at G1/S and G2/M, may be more dependent on ATR to resolve DNA damage before a cell cycle transition generates potentially lethal mutations and mitotic catastrophe [1,4,5]. Thus, in certain tumors ATR kinase signaling may mediate repair processes that limit cancer cell killing to a greater extent than normal tissue [6]. ATR is therefore a potential therapeutic target whose inhibition may potentiate the effects of DNA damaging chemotherapies and IR. Currently, there are four small molecule ATR inhibitors (ATRi) in various phases of clinical development: AZD6738 (ceralasertib), M6620 (VX-970, berzosertib), BAY-1895344 (elimusertib), and M4344 (VX-803) [7].

There is extensive preclinical evidence demonstrating the efficacy of ATRi, but much is still unknown as the class progresses through development [1,7]. A looming question for the drug class concerns whether concomitant treatment with DNA damaging chemotherapies shift the therapeutic window of the targeted tumor site without increasing toxicity in off-target normal tissues to intolerable levels [8]. This is also relevant with DNA damage from targeted stereotactic radiation treatments where IR has the capacity to non-discriminately damage healthy tissues proximal to the targeted tumor site, with tissues having unique tolerance thresholds to IR [9,10]. The addition of an ATRi to concomitant IR or chemotherapy may increase the frequency and severity of adverse events [11].

The pharmacokinetics (PK) and pharmacodynamics (PD) of ATRis have not been described in detail. The major PK and PD factors influencing the therapeutic window are ATRi tissue distribution and tissue specific sensitivity of DNA damage related toxicity [12,13]. Comparative PK and PD characterization of the different ATRi may allow optimal development of each member and impact clinical treatment strategies.

AZD6738 is an orally available small molecule ATRi in clinical development and is currently being studied in at least 32 early clinical trials (Clinicaltrials.gov accessed date 10/1/21). Despite extensive evidence of AZD6738 radio- and chemo-sensitization in preclinical studies, little is known about the PK, including the dose linearity and bioavailability, which may impact a multitude of effects related to efficacy or safety [5,14].

Also relevant is that IR can suppress immune responses and AZD6738 has been found to attenuate this and produce durable anti-tumor immune responses following IR in mouse models [15–18]. AZD6738 promotes CD8+ T cell anti-tumor responses and attenuates IR-induced tumor cell PD-L1 expression [15,16,19]. There is limited evidence suggesting these effects extend to the entire ATRi drug class but the exact relationship of these immune effects with ATRi exposure is unknown [20].

It is likely that the efficacy, toxicity, and immune effects of AZD6738 are dependent on relevant tissue concentrations. We therefore evaluated the PK of AZD6738, covering oral bioavailability, dose linearity, and tissue distribution.

## 2 MATERIALS AND METHODS

### 2.1 Chemicals and Reagents

**Figure 1.**
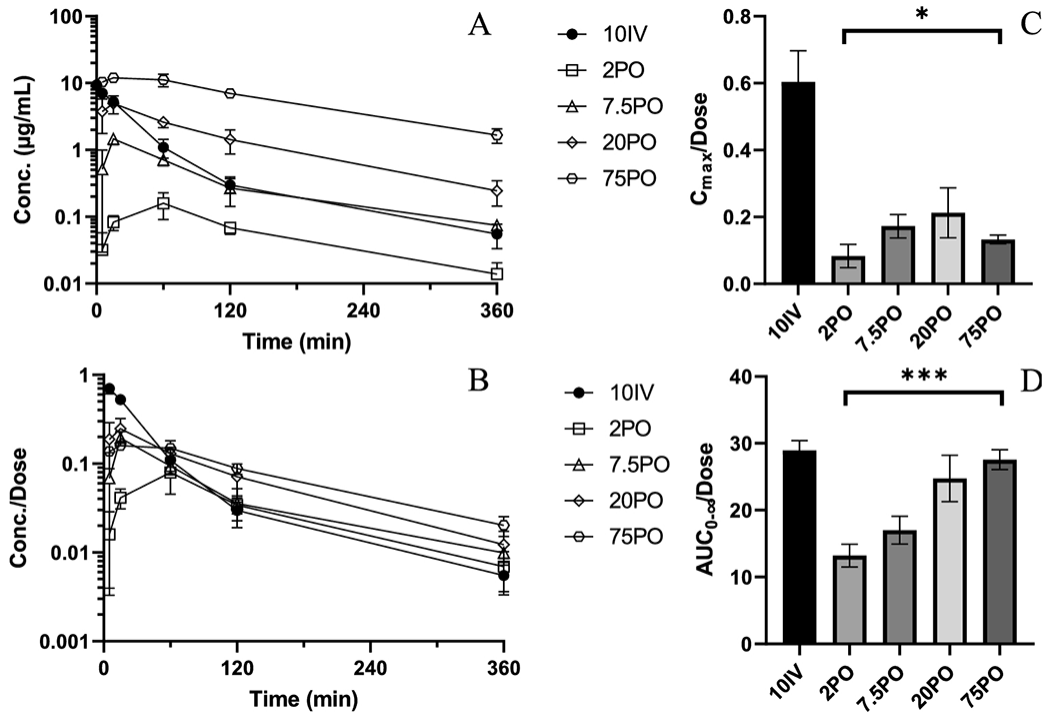
PK and NCA of AZD6738 from bioavailability and dose linearity studies. A) Mean plasma concentration versus time profiles for 10IV (●), 2PO (○), 7.5PO (◇), 20PO (△), 75PO (□). B) Mean plasma concentrations normalized by administered dose versus time profiles C) Dose-normalized C_max_ (ANOVA test of PO treated groups, p=0.0395) D) Dose-normalized AUC_0-∞_ (ANOVA test of PO treated groups, p=0.0002). Error bars represent ± SD for concentrations and ± SEM for AUC.

AZD6738 was purchased from AdooQ Bioscience (Irvine, CA) and [^2^H_4_]-AZD6738 (Suppl.Figure 1) was custom synthesized and purchased from ALSACHIM (Illkirch-Graffenstaden, France). Water and methanol (both HPLC grade), formic acid, DMSO (used for vehicle formulation) were obtained through Fisher Scientific (Fairlawn, NJ). DMSO used for dissolving standards used in analytical portions was obtained from Sigma-Aldrich (St. Louis, MO), propylene glycol was obtained from MP Biomedicals, LLC (Solon, OH).

### 2.2 Mice

Specific pathogen-free female Balb/c mice (5-7 weeks of age) were purchased from Charles River (Wilmington, MA). Mice were allowed to acclimate to the University of Pittsburgh Animal Facility for at least 1 week before studies were initiated. To minimize exogenous infection, mice were maintained in microisolator cages and handled in accordance with the Guide for the Care and Use of Laboratory Animals (National Research Council, 2011) and on a protocol approved by the University of Pittsburgh IACUC. Ventilation and airflow in the animal facility were set to 12 changes/h. Room temperature was regulated at 72 ± 4 °F and the rooms were kept on automatic 12-h light/dark cycles. The mice received Prolab ISOPRO RMH 3000, Irradiated Lab Diet (PMI Nutrition International, Brentwood, MO) and water *ad libitium.* Mice at each time point were stratified by body weight (and secondly by tumor size if applicable). Throughout all studies, mice were routinely weighed and monitored for changes in health.

### 2.3 Dose Linearity and Bioavailability

Mice (N=3 per time point) were dosed with 10 mg/kg AZD6378 IV with a 30 s bolus in the tail vein or 2.0, 7.5, 20, or 75 mg/kg AZD6738 PO was administered by gavage. The vehicle for all groups was 10% DMSO, 40% propylene glycol, and 50% dH_2_O. Sample collection time points were 5, 15, 60, 120, 360 and 1440 min. Vehicle controls were included at 5 and 1440 min. Mice were euthanized by CO_2_ inhalation and the following tissues collected: blood, liver, kidney, lung, skeletal muscle, and brain. Blood was collected by cardiac puncture using EDTA anticoagulated syringes, transferred to microcentrifuge tubes and centrifuged at 12,000 × g for 4 min to separate plasma and red blood cells (RBCs). Pooled urine and feces were collected over ice from mice in the 1440 min groups housed in metabolic cages.

### 2.4 Extensive Tissue Distribution Studies

Mice were injected subcutaneously on the right flank with 1x10^6^ CT26 cells, a murine colorectal carcinoma cell line from BALB/c mice,obtained from ATCC (Manassas, VA). Cells were cultured in RPMI-1640 medium with L-glutamine (BioWhittaker Inc., Walkersville, MD), containing 10% heat-inactivated fetal bovine serum and 100 units of penicillin/mL and 100 μg/mL of streptomycin (Biofluids, BioSource, Rockville, MD) in an incubator with 95% air, 5% CO_2_, and 95% humidity at 37 °C. Cells were verified and checked for mycoplasm by IDEXX BioAnalytics (Westbrook, ME). Implantation and tumor growth were monitored twice weekly with a digital caliper. When tumors were approximately 200 mm^3^, mice were stratified into time groups (N=3) primarily using body weight and secondarily using tumor size. Mice were administered 75 mg/kg PO through oral gavage. Sample collection time points were 5, 15, 30, 60, 120, 240, 360, 960, and 1440 min and vehicle controls were included at 5 and 1440 min. At each time point, mice were euthanized by CO_2_ inhalation and the following tissues collected: blood, liver, kidney, spleen, lung, skeletal muscle, brain, heart, fat, tumor, small intestine (flushed with PBS), esophagus (flushed with PBS), spinal cord, thymus, draining lymph node, non-draining lymph node, and bone marrow. Bone marrow was obtained by flushing both femurs from each mouse with PBS, followed by centrifugation at 12,000 x *g* and removal of the supernatant. Protein analysis of the resuspended pellet was conducted using the Bio-Rad protein assay following the manufacturer’s instructions with bovine serum albumin as the standard. Blood was processed to obtain plasma and urine and feces were collected as described above.

### 2.5 Bioanalysis

An LC-MS/MS assay was implemented on a system consisting of an Agilent (Palo Alto, CA) 1290 Infinity II Autosampler and Binary Pump and a SCIEX (Concord, ON, Canada) 6500+ mass spectrometer to quantitate AZD6738 in plasma, and tissues. This assay was based on a previously validated assay and several modifications were made to increase sensitivity and improve robustness [21]. This included the use of [^2^H_4_]-AZD6738 as an isotopic internal standard, changes to calibration standard and QC ranges, sample preparation, alterations to the HPLC gradient method, and the addition of semi-quantitation of reported major sulfone and sulfoxide metabolites (Suppl.Figure 1) as identified previously *in vitro* with human liver microsomes (HLMs) [22].

Mass spectrometric conditions were determined using infusion of neat standards and were optimized as follows: 30 L/h curtain gas, 5,500 ion spray voltage, 500 °C temperature, 70 L/h ion nebulizer gas 1 and auxiliary gas 2, high collision cell gas, 60 V declustering potential, 4 V entrance potential, 40 V collision energy, 15 V collision exit potential. MRM channels were 413.2>334.0 for AZD6738 and 417.2>338.2 for the [^2^H_4_]-AZD6738 with dwell times of 0.1 s. Metabolite channels of 414.2>264.2 398.2>335.2 were monitored for the sulfone metabolite and sulfoxide metabolites, respectively.

Chromatographic separation was still achieved using a Phenomenex (Torrance, CA) Synergi Polar-RP 80A column (4 μm, 2 mm × 50 mm) at ambient temperature with a gradient mobile phase program consisting of mobile phase solvent A (water with 0.1% formic acid (*v/v*)) and mobile phase solvent B (methanol with 0.1% formic acid (*v/v*)). A gradient HPLC method was used with beginning with an initial condition of 65% mobile phase A pumped at 0.3 mL/min that was decreased to 5% over the course of 3.5 min. This was held until 4.0 when the composition was maintained, and the flow rate increased to 1.0 mL/min until 4.5 min. At 4.6 min the composition was returned to initial conditions and pumped at 1.0 mL/min for an additional 1.4 min resulting in a total run time of 6.0 min. Flow was diverted to waste between 0 to 1.5 min and 4 to 6 min.

Duplicate standard curves with calibrators consisting of 0.3, 1, 3, 10, 30 100, 300, and 1,000 ng/mL, in addition to blank plasma, were prepared fresh on days of analysis using control Balb/c mouse plasma (Innovative Research, Novi, MI). QCs were prepared in bulk and stored at −80 °C, with concentrations of 0.8 (QCL), 20 (QCM), and 800 ng/mL (QCH). Samples were prepared by adding 10 μL of 0.05 μg/mL of [^2^H_4_]-AZD6738 to each 50 μL sample followed by the addition of 250 μL of methanol for protein precipitation. Samples were then vortexed for 1 minute, centrifuged for 10 min at 13,500 x g, and 100 μL of the resulting supernatant transferred to HPLC vials. The injection volume was 3 μL. Retention times were 3.2 min for both AZD6738 and internal standard. Curve fitting was accomplished through linear regression with 1/y^2^ weighting. Semi-quantitation of metabolites was accomplished by reporting IS normalized metabolite response.

To validate the assay, a triplicate standard curve in plasma with QCs (N=6, per level) was analyzed to determine analytical accuracy and precision. Assay performance in matrices of RBC and tissue homogenates were determined by spiking control samples to the 20 ng/mL QCM level in replicates of 4 and determining their accuracy based on a calibration curve constructed in mouse plasma. Analyte recovery and matrix effect of plasma, as well as tissue and tumor homogenate, were analyzed in plasma by comparing QCM samples with both QCM level spiked neat and plasma background samples (N=4).

For tissue and tumor analysis, samples were homogenized with 3 parts PBS (*v/g*) and further diluted with control plasma and concentrations determined from calibration curves prepared in control plasma.

Due to a lack of reference standards, the assay was not optimized or validated for quantitating sulfoxide and sulfone metabolites, nor were metabolite stability and linearity in response established. Noncompartmental analysis (NCA) of metabolites was accomplished through semi-quantitative means by reporting concentrations as the IS normalized value of their peak area and AUC as IS normalized values per unit time. Inclusion of metabolite data was based on discernable peak area greater than that in vehicle treated plasma.

**Figure 2.**
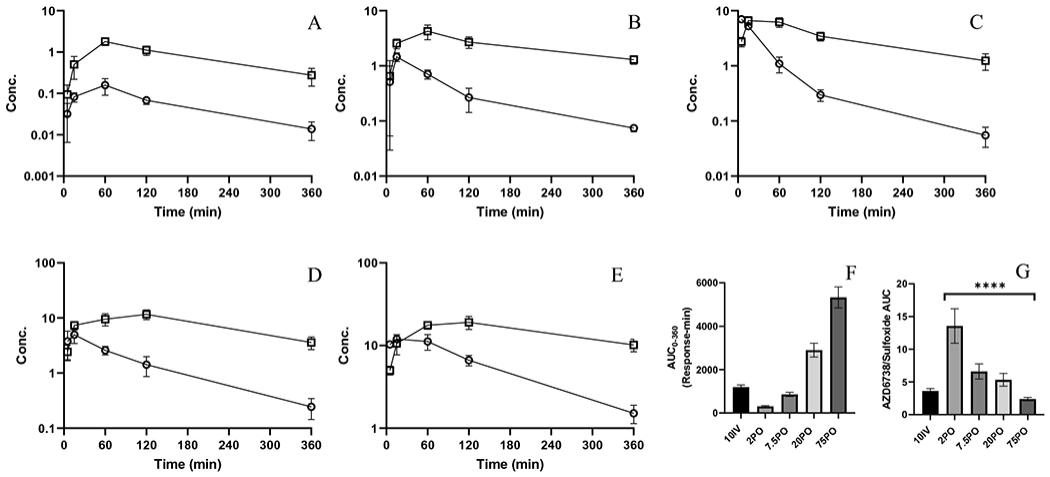
Mean (SD) plasma AZD6738 (○, μg/mL) and sulfoxide (□, IS normalized response) profiles A) 2 mg/kg PO; B) 7.5 mg/kg PO; C) 20 mg/kg PO; D) 75 mg/kg PO; E) 10 mg/kg IV; F) Sulfoxide metabolite AUC0-360 G) Metabolic ratios constructed from metabolite and parent AUC0-360 (ANOVA test of PO groups, p<0.0001). Error bars represent ± SEM.

The accuracy and precision of a triplicate standard curve and QCs met the acceptance criteria for FDA bioanalytical method validation with accuracies of calibrators and QCs ranging between 89.1 to 114.3% with precisions <6.1% (see Suppl.Table 1) [23]. A representative chromatogram of the AZD6738 and IS response of an LLQ sample can be seen in Suppl.Figure 2. Analyte recovery was 109% (CV 2.5%), indicating no loss during protein precipitation and matrix effect caused by plasma was −10.7% (CV 2.0%). Both recovery and matrix effect matched the previously validated assay [21].

### 2.6 Pharmacokinetic Analysis

#### 2.6.1 Noncompartmental Analysis

Noncompartmental (NC) PK was determined using GraphPad Prism (San Diego, CA) using the linear trapezoidal method and Bailer method of AUC determination for estimates of exposure in plasma, RBC, tumors, and tissues [24]. PK parameters or analysis requiring dose were adjusted for the actual dose as determined using measurements of dosing solution. Dose-normalized plasma exposures, C_max_ and AUC, of PO treated groups of the dose linearity studies were compared using ANOVA tests to determine dose linearity.

Tissue partition coefficients (P_Tissue_) and RBC partitioning (P_RBC_) were calculated using Eq. (1) and tested among PO treated groups using ANOVA tests.

For analysis of urine and feces from 1440 min groups, urine was diluted at least 1:10 in control plasma and feces was homogenized 3:1 in PBS. The resulting concentrations were multiplied by the volume or weight to convert to a total amount and then divided by 3 to account for the number of mice per cage. These amounts were then divided by the average total amount of drug administered to calculate the percent excreted by urine and feces, see Eq. (2). Renal clearance (Cl_R_) was calculated by dividing the excreted amount by the plasma AUC, see Eq. (3) [25].

#### 2.6.2 Metabolite Analysis

When available, plasma metabolite data was used to determine semi-quantitative exposure as well as metabolic ratios, using Eq. (4). The MR among PO treated groups in the dose linearity studies were compared using ANOVA testing. Metabolite presence was also analyzed in urine and feces.

#### 2.6.3 Intestinal Contribution to Oral Bioavailability

The contribution of gut to oral bioavailability was estimated by calculating the contribution of liver processes to bioavailability from IV data and stripping this contribution from the observed bioavailabilities [26,27]. Assuming IV drug clearance was restricted to hepatic processes, the predicted bioavailability based solely on liver processes, F_H_, was calculated from IV PK. The liver extraction ratio (E_H_), see Eq. (5), was constructed using literature values of hepatic blood flow (Q_H_) in conjunction with study specific PK items including the fraction of drug excreted in urine (*fe*) and total blood clearance (Cl_Blood_) as seen in Eq. (6) [26,27]. *Fe* was derived from the amount of drug measured in urine (*Ae*) and the total amount of drug administered, see Eq. (7). Cl_Blood_ was defined by multiplying the observed plasma clearance (Cl_plasma_) by the ratio of plasma exposure (AUC_plasma_) to total blood exposure (AUC_Blood_), see Eq. (8). AUC_Blood_ was calculated using hematocrit (HCT) to construct total blood exposure contributions from exposure in RBC (AUC_RBC_) and plasma, see Eq. (9).

Observed *in vivo* bioavailabilities (F_observed_) contain cumulative contributions from both absorption, gut, and hepatic processes, F_A_, F_G_. and F_H_ respectively (see Eq. (10) [26]. To isolate and identify the gut contribution, absorption was assumed to be uniform and complete (F_A_=1) for all PO groups. The predicted hepatic contribution was subtracted from the observed *in vivo* bioavailability to yield the gut contribution to bioavailability for each PO treatment group, see Eq.(11).

#### 2.6.4 Compartmental Model

A unified compartmental PK model was developed using ADAPT5 [28] to fit the data, to fit the data. Naïve-pooled concentration-time data from each study was used to estimate PK parameters that were shared between each treatment group. Model performance was determined and reported using parameter CV%, R^2^, AIC (Akaike information criterion), as well as visually inspected with standardized residual plots. Drug that left the absorption compartment (through pathway k_na_) but did not enter the systemic circulation (presystemically cleared drug) was collected in a mock compartment without elimination. The amount of drug ultimately collected in this compartment allowed for calculation of the bioavailability. The developed model was then used to simulate expected plasma concentrations in the extensive PK study. Separately, tumor and plasma time concentration data from the extensive PK study was used to extend this model to add an uncoupled tumor compartment [29].

### 2.7 Plasma Protein Binding

Plasma protein binding of AZD6738 was determined using rapid equilibrium dialysis (RED) devices (Thermo Fisher Scientific, Waltham, MA). Freshly collected Balb/c mouse plasma was spiked in replicates of N=3 at 1,000 and 10,000 ng/mL using stock solutions with < 0.1% organic solvent in final samples, for a 6 h incubation.

## 3 RESULTS

### 3.1 Dose Linearity and Bioavailability

#### 3.1.1 Noncompartmental Analysis

Plasma concentrations versus time profiles for all dose linearity and bioavailability studies are depicted in Figure 1A and PK parameters are detailed in Table 1. IV administered mice displayed a biphasic concentration-time profile. The C_max_ for PO administered mice were observed at 15 min except in the 2 mg/kg group where it occurred at 60 min. Non-linearity was observed with non-superimposable dose-normalized concentrations (Figure 1B) as well C_max_ and AUCs (Figure 1C–D). This resulting non-linearity corresponded to a more than two-fold increase in the observed bioavailability between the 2 and 75 mg/kg doses. Observed half-lives ranged from 94.4 to 130 min, without any apparent trend with the doses. Both apparent clearance (Cl/F/kg) and volume of distribution (V_d_/F/kg) decreased by approximately 50% between the lowest and highest PO doses. The apparent clearance and volume of distribution observed in the 75 mg/kg group approximated that of the 10 mg/kg IV group.

Renal excretion of unaltered AZD6738 was a minor route of elimination with <4% of dose being recovered across all groups. Approximately 15% of dose was excreted unchanged in the feces after IV dosing and 9.2 to 15.7% after PO dosing with no discernable trend as a function of PO dose.

**Figure 3.**
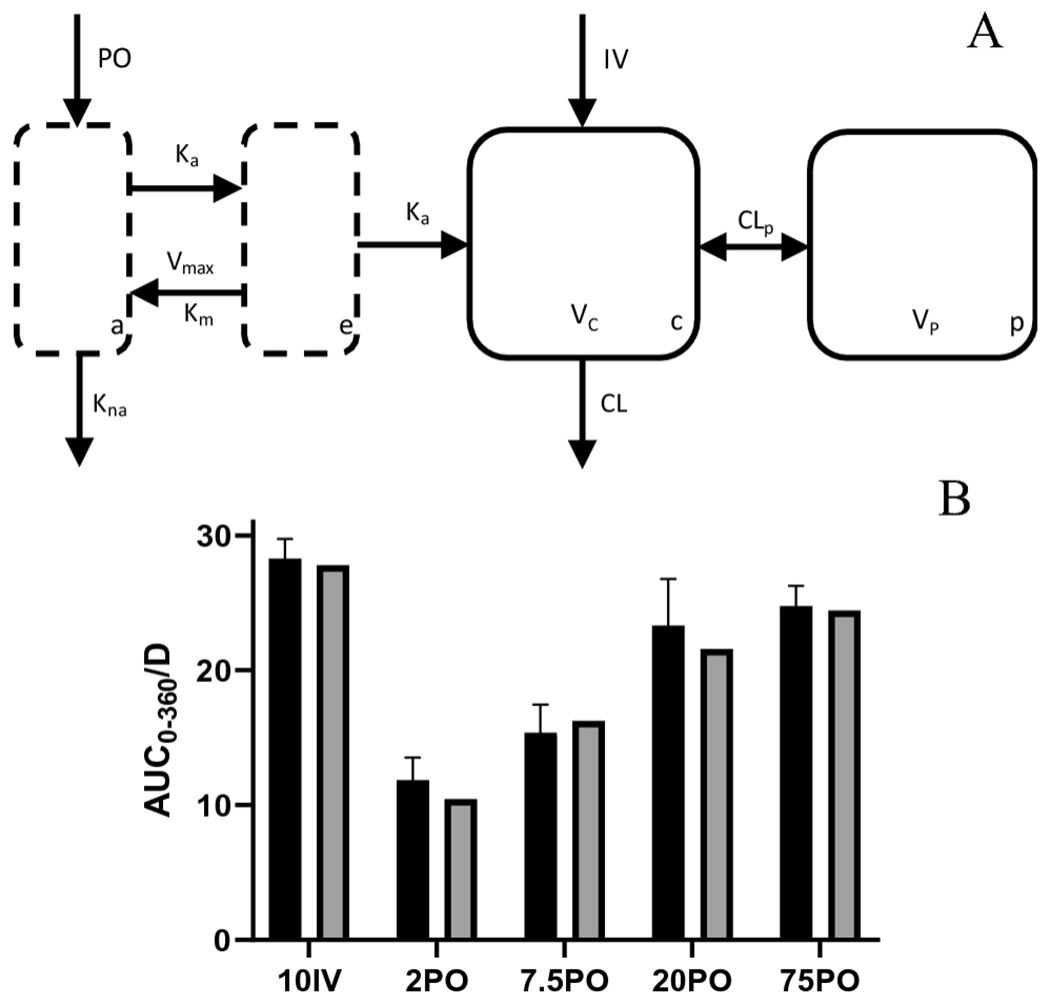
Unified compartmental PK model (A) model structure showing saturable first-pass metabolism absorption through gut lumen and enterocyte compartments that lead to a two-compartment model; (B) AUC_0-360_ observed (±SEM) in dose linearity and bioavailability groups (solid black) and compartmental model prediction (solid grey).

The T_max_ and elimination half-life of tissues mirrored plasma from their respective doses (Suppl.Figure 3A–E). RBC and tissue exposure (Suppl.Table 2) were normalized to form partition coefficients to identify any additional contributions to non-linearity (Suppl.Table 3). Notably, RBC partitioning appeared to increase with the PO dose (Suppl.Figure 4A). Apparent liver partitioning appeared to decrease with increasing PO dose Suppl.Figure 4B). This effect diminished when liver exposure was normalized by dose (data not shown), indicating liver exposure is more defined by the administered dose than plasma concentrations. Partitioning to kidney, selected to represent rapidly perfused tissue, was generally uniform across all groups (Suppl.Figure 4C). Partitioning to lung was similar among PO groups but approximately two-fold higher in PO groups compared to the IV group (Suppl.Figure 4D). Partitioning to muscle, selected as a poorly perfused tissue, did not deviate between treatment groups (Suppl.Figure 4E). Penetration into the brain was limited with uniform partitioning across groups (Suppl.Figure 4F).

#### 3.1.2 Metabolite Analysis

The AZD6738 sulfoxide metabolite was detected in plasma over 360 min (Suppl.Figure 5A). Concentration-time profiles (Figure 2A–E) reveal metabolite terminal profiles largely mirroring parent AZD6738 indicating formation rate-limited metabolite elimination. NCA revealed that sulfoxide exposure increased as dose increased (Table 2 and Figure 2F). Metabolic ratios (MR) revealed drastic decreases as PO dose increased (Figure 2G), indicating metabolic pathway saturation as dose increased. The MR in IV treated mice, which conventionally is largely limited to hepatic metabolism, was relatively low and approximated that found in higher dose PO groups. While the sulfone metabolite was not detected in plasma, it was detected with the sulfoxide in both feces and urine (Suppl.Figure 5B).

#### 3.1.3 Intestinal Contribution to Oral Bioavailability

The hepatic contribution to bioavailability based on IV PK data predicted an F_H_ of 0.633, see Suppl.Table 4. This was then used to discern the intestinal metabolism component of first-pass metabolism using observed bioavailabilities to produce an F_G_ of 0.681 for the lowest PO dose and increasing with dose to a maximum of 1.42 (Suppl.Table 5). F_G_ values greater than 1.0 indicate saturation of liver metabolism in addition to saturation of intestinal metabolism. The assumption of uniform and complete absorption (F_A_) was validated by the uniform presence of unaltered drug in feces across doses and administration routes.

#### 3.1.4 Compartmental Model

As determined by NCA, bioavailability increases with PO dose, suggesting saturation of metabolism and/or efflux transport. To accommodate this, different model structures were evaluated, resulting in the one depicted in Figure 3A, where the nonlinearity was captured by a pre-systemic saturable efflux/metabolism component (denoted using V_max_ and K_m_). The inclusion of an efflux-like mechanism was necessary to accommodate the later T_max_ observed in the 2 mg/kg PO group. Model structures with separate metabolic and efflux processes were not identifiable. The model fit the data well, see Suppl.Table 6 and Suppl.Figure 6A–J for estimated PK parameters and model fit. Model-based estimates were similar to NCA values for AUC (Figure 3B) as well as bioavailability (Suppl.Table 7) and well captured the non-linearity. For equations of the final model structure, see aEquations 12–15.

### 3.2 Extensive Tissue Distribution Studies

#### 3.2.1 Noncompartmental Analysis

The plasma concentration-time profile and PK in tumor bearing animals agreed with the PK of the 75 mg/kg non-tumor bearing group (Table 1 and Suppl.Figure 7A). In most tissues, the drug distribution appeared to be perfusion-rate limited and rapid equilibrium was reached with plasma except for fat, tumor, and bone marrow, each of which reached later peak concentrations at 30 min and appeared diffusion-rate limited in profile (Table 3 and Suppl.Figure 7B–R). Most tissues experienced exposure at least as great as plasma except for brain, spinal cord, and bone marrow (Suppl.Figure 8A–B). All samples were above the 0.3 ng/mL LLQ at 1440 min except brain and bone marrow. Metabolite data mirrored that found in the dose-linearity and bioavailability studies (Suppl.Table 8 and Suppl.Figure 9).

#### 3.2.2 Compartmental Model

The addition of the uncoupled tumor compartment to the plasma-based model (Suppl.Figure 10A) performed adequately with the model fitting the data well (Suppl.Table 9 and Suppl.Figure 10B–E). Underlying equations for the final model structure can be seen a Equations 16–20.

### 3.3 Plasma Protein Binding

Plasma protein binding was moderate, with unbound fractions of 31% and 35% at 1 and 10 μg/mL respectively (Suppl.Table 10).

## 4 DISCUSSION

We aimed to determine AZD6738 distribution in tissues that have a potential impact on efficacy, toxicity, and immunological response. Initial efforts established the relationship between dose and systemic exposure. These studies demonstrated a significant non-linearity during absorption due to saturable first-pass metabolism in mice. Dose-dependent bioavailability is relatively common and has been observed for other drugs both preclinically [30] and clinically [31]. While several mechanisms may explain this phenomenon, for AZD6738 evidence was found supporting saturable first-pass metabolism in both the gut and the liver.

The saturable mechanisms were reflected in a compartmental PK model, and while it does not claim to represent anatomical and physiological absorption processes, it did mathematically capture the observations. The model accommodated saturable removal of drug presystemically through cyclical metabolism and efflux mechanisms. Minimal differences in unaltered excreted drug between IV and PO treated mice suggests that all drug that did not enter the central compartment was metabolized presystemically with biliary excretion as a possible additional mechanism of systemic clearance.

The limited renal clearance and high bioavailability of AZD6738 observed in mice highlights the large role of metabolism in both pre-systemic and systemic clearance. Qualitatively, both the sulfone (urine and feces) and sulfoxide (plasma, urine and feces) metabolites, previously identified *in vitro* using HLMs, were identified as metabolic products of AZD6738 in mice. The inverse relationship of the sulfoxide metabolic ratio with dose suggested a saturable pre-systemic mechanism explaining the observed dose-dependent bioavailability. Metabolite analyses could be used clinically to confirm saturable metabolism, as exemplified in a study of the estrogen receptor degrader AZD9496 [31].

Clinical translation of our findings would need to consider species differences in enzymes as well as gut wall and liver expression differences [32,33]. It is unknown which enzymes in murine gut and liver contribute to AZD6738 metabolism, although previous studies in HLMs identified oxidative deimination to a primary sulfoxide metabolite by CYP2C8/2J2 followed by conversion into a secondary sulfone metabolite formed by CYP2J2/3A [22]. CYP2C8 is an important metabolizing enzyme for multiple drugs, and is highly expressed in the liver with limited expression in the small intestine [34,35]. CYP2J2 expression is primarily expressed in the intestines of both humans and animals, where it has been found to clinically impact first-pass metabolism of the antihistamine astemizole [36,37]. CYP3A, implicated in producing the sulfone metabolite in HLMs, can effect both intestinal and hepatic metabolism [38]. CYP3A substrates (e.g. cyclosporin) are often also P-glycoprotein (P-gp) substrate [39]. Interplay between metabolizing enzymes and transporters, such as CYP3A and P-gp, can act synergistically to limit oral bioavailability, as exemplified with paclitaxel in mice where substantial limitations in bioavailability were found to be largely due to this relationship [40]. Additionally, substrates for CYP2C8 metabolism have also been found to overlap with P-gp, as noted with the anti-diarrheal medicine loperamide [41]. Whether AZD6738 is a substrate for P-gp is unknown. Indirect evidence of AZD6738 as a transporter substrate includes the earlier T_max_ at higher PO doses that may demonstrate saturation of transporter efflux in the gut and the limited CNS partitioning where transporters can limit distribution.

Partition coefficients for the tissues studied in the dose linearity and bioavailability studies were largely uniform across PO doses. As such, the partition coefficients identified in the extensive PK study should be applicable to any dose except for tissues with significant pre-systemic exposure such as the esophagus, small intestine, and liver. These tissues, as well as highly perfused tissues, experienced the highest drug concentrations and, as a result, may be more susceptible to toxicity from concomitant IR or chemotherapy due to ATR inhibition. This is particularly relevant to head and neck cancer where severe mucositis is caused by treatment with IR and the addition of an ATRi may increase the incidence of this toxicity [42]. Fat and bone marrow, both poorly perfused, demonstrated a delayed C_max_ compared to plasma and displayed diffusion limited distribution. Brain and spinal cord had the lowest exposures, with similar partition coefficients of approximately 0.05. This amount of drug present may actually be from the vascularized portion of the brain, which is less than 5% of total brain volume, and suggests that almost no drug penetrates the blood-brain barrier [43]. The evidence for limited CNS distribution agrees with earlier preclinical reports showing AZD6738 brain penetration which achieved an approximate 0.2 μg/mL concentrations 1 h after 25 mg/kg IP administration [44]. While they did not report complete AUC, the brain to plasma concentrations ranged from 0.26 to 0.63, which are approximately 4 to 12-fold higher than values we reported in the extensive tissue distribution study. Notably, quantitation from these studies appears to have been performed semi-quantitatively without internal standard or validation.

Within the dose linearity studies, there was a potential non-linearity in RBC observed by increases in RBC partitioning as dose increased. Saturable protein binding is unlikely to contribute to this because plasma protein binding at relevant plasma concentrations showed only modest increases in unbound drug with increasing concentrations. Additionally, no other impact was observed on partition coefficients or renal clearance. The extensive PK study revealed a RBC partitioning which did not fit the observed non-linear trend.

Tumor exposure was approximately 75% that of plasma. The delay in peak tumor concentrations compared to plasma may reflect poor tumor vascularization that results in diffusion rate limited distribution. Concentrations were greater than the cell based IC_50s_, which are generally reported below 413 ng/mL (1,000 nmol/L), until at least 360 min [14]. The tumor kinetics were adequately captured with the uncoupled tumor model. Distribution into lymph node, an organ of immunological importance, was limited and similar to tumor, with a negligible difference in exposure between draining and non-draining lymph nodes.

The murine doses studied are comparable to human doses based on BSA-equivalency [45]. The PO doses of 2, 7.5, 20, and 75 mg/kg in mice correspond to, 10, 37, 98 and 366 mg in humans, and clinically studied doses range from 20 to 240 mg BID or QD [7] (NCT03022409, NCT04239014 NCT03330847, NCT03334617, NCT03669601, NCT02223923). Although there is limited PK reported from these trials, there are some preliminary results. A single agent AZD6738 trial investigating 20 to 240 mg BID, reported approximate dose proportionality in exposure from 80 to 240 mg but greater than proportional increases from 20 to 40 mg [46]. Possibly, the non-linear absorption observed in our mouse studies occurs at clinical doses of 20-80 mg. A phase I trial of AZD6738 BID in combination with olaparib and durvalumab, declared dose proportionality from 60 to 240 mg (NCT02264678) [47]. This study found substantial but reversible dose-dependent decreases in peripheral monocytes, which originate from the bone marrow, an organ with substantial AZD6738 exposure in our studies [48]. Additional clinical studies reported thrombocytopenia as the dose limiting toxicity (DLT), with additional toxicities seen from decreases in monocytes and T-cells [49,46]. A PK-PD model developed to describe the relationship of these toxicity effects with PK found monocyte and thrombocyte decreases occurred at concentrations of 2.5 μg/mL and increased up to 10 μg/mL [49]. These clinical concentrations were achieved in mice beginning at 20 mg/kg AD6738. Future reports on monocyte and thrombocyte profiles in mice could clarify whether mice recapitulate these clinical observations.

## 5 CONCLUSION

The results from these studies highlight the importance of early and thorough description of PK during drug development and negate common assumptions that dose and exposure change proportionally. Preclinical studies of novel agents, including AZD6738, often suggest that dose and exposure are interchangeable when relating either to an observed effect. Describing and understanding the relationship between dose and exposure can bridge either parameter to observed PD responses. The PD of AZD6738 has been studied in a variety of preclinical settings and the results reported here offer a direct link of dose and exposure allowing more accurate, albeit more complex, relationship of dose and PD response.

Any ATRi related effect of toxicity or efficacy are ultimately reliant on exposure at the site of action and the sensitivity of ATR inhibition at a respective tissue. The exposure of drug at the site of action was found to be unique for most tissues of relevance to toxicity, efficacy, or immunologic importance. This is highlighted by the limited distribution of AZD6738 to brain and spinal cord, which will likely limit its usefulness as an agent against primary or metastatic CNS tumors. The results reported here comprehensively describe the PK of relevant tissues and facilitate future descriptions of tissue specific sensitivity. They also provide a framework for directly comparing exposure to other ATRi by contrasting partition coefficients and tissue concentrations. Understanding ATRi specific tissue distribution and tissue sensitivity, relative to tumor exposure, may result in disparate therapeutic indices that can guide clinicians in selecting a specific ATRi to minimize the toxicity and maximize the efficacy of a particular therapeutic regimen.

## Supplementary Material

1774452_Equation

## Figures and Tables

**Table 1.** Noncompartmental AZD6738 PK parameters

Route Dose (mg/kg)	IV 10	PO 2.0	PO 7.5	PO 20	PO 75	p-value^b^	PO-ext^f^ 75
Actual dose (mg/kg)	11.7	1.92	8.8	23.4	90.5		88.4

C_max_ (μg/mL)	7.04 (0.93)	0.160 (0.069)	1.46 (0.26)	4.96 (1.50)	12.0 (1.0)		14.1 (1.7)
C_max_/dose^a^	0.604 (0.093)	0.0829 (0.034)	0.172 (0.035)	0.212 (0.075)	0.133 (0.013)	0.0395	0.160 (0.019)
T_max_ (min)	5	60	15	15	15		15
AUC_0-360_ (μg/mL•min)	330 (17)	22.8 (3.3)	130 (18)	545 (81)	2,241 (135)	0.0002	2,371 (185)
AUC_0-360_/dose^a,c^	28.3 (1.5)	11.8 (1.7)	15.4 (2.1)	23.3 (3.5)	24.8 (1.5)		26.8 (2.1)
AUC_0-∞_ (μg/mL•min)	337	25.4	144	578	2,495		2,371 (185)
AUC_0-∞_ /dose^a^	28.9	13.2	17.0	24.7	27.6	0.0002	26.8 (2.1)
F^d^	-	0.457	0.588	0.855	0.952		
CI (mL/min/kg)^e^	34.5	75.7	58.8	40.4	36.3		37.3
V_d_ (L/kg)^e^	4.92	11.4	11.0	5.50	6.06		5.66
Half-life (min)	98.6	104	130	94.4	116		105
0-24h urine (% dose)	1.91	2.51	3.07	3.61	3.06		3.19
CI_R_ (mL/min/kg)	0.661	1.97	1.88	1.53	1.14		1.19
0-24h feces (% dose)	13.9	15.7	13.5	16.1	9.22		8.58

Values represent the mean and error (SD for C_max_, SEM for AUC)

a: calculated using exact dose

b: ANOVA test of PO routes only

c: % extrapolated ranges from 0.008 to 10.4%

d: Based on AUC_0-∞_

e: Apparent parameter for initial 4 PO doses 2, 7.5, 20, and 75 mg/kg

f: Plasma PK parameters for the tissue distribution study

**Table 2.** AZD6738 Sulfoxide Metabolite Semi-Quantitative NCA.

Route Dose (mg/kg)	IV 10	PO 2.0	PO 7.5	PO 20	PO 75	p-value^a^
C_max_	6.64 (1.19)	1.79 (0.34)	4.25 (1.26)	11.7 (2.29)	19.1 (3.45)	-
C_max_/dose	0.570 (0.102)	0.931 (0.179)	0.502 (0.149)	0.500 (0.098)	0.211 (0.038)	0.0252
T_max_ (min)	15	60	60	120	120	-
AUC_0-360_	1,191 (107)	309 (41)	859 (96)	3,897 (319)	5,327 (486)	-
AUC_0-360_/dose	102 (9)	161 (21)	101 (11)	124 (14)	58.9 (5)	0.0001
MR AUC_0-360_	3.61 (0.37)	13.6 (2.6)	6.60 (1.16)	5.32 (0.98)	2.38 (0.26)	<0.0001
Half-life (h)	136	113	186	141	186	-

Values are reported as mean and error (SD for C_max_ and SEM for AUC)

a. ANOVA testing and reported p-value limited only to PO data

**Table 3.** Extensive PK Study Tissue PK and Partition Coefficients

Tissue	C_max_ (μg/mL)	T_max_ (min)	AUC_0-t_^a^ (μg/mL•min)	AUC_0-∞_^a^ (μg/mL•min)	Partition Coefficient

Plasma	14.1 (1.7)	15	2,371 (185)	2,371 (185)	-
RBC	13.2 (3.3)	15	1,662 (149)	1,663 (149)	0.701 (0.083)
Liver	114 (30)	15	17,596 (1212)	17,603 (1212)	7.42 (0.77)
Kidneys	39.2 (8.7)	15	6,563 (335)	6,564 (335)	2.77 (0.26)
Spleen	14.2 (1.6)	15	2,112 (158)	2,113 (158)	0.891 (0.096)
Lungs	17.4 (3.5)	15	2,527 (191)	2,528 (240)	1.07 (0.13)
Heart	19.4 (5.3)	15	2,735 (212)	2,738 (212)	1.15 (0.13)
Fat	9.2 (8.1)	30	975 (186)	976 (258)	0.412 (0.114)
Muscle	12.5 (6.9)	15	2,473 (182)	2,475 (182)	1.04 (0.11)
Brain	0.854 (0.515)	15	126 (11)	126 (11)	0.0532 (0.0063)
Tumor	10.1 (2.3)	30	1,774 (224)	1,777 (224)	0.750 (0.111)
Small Intestine	204 (49)	15	19,882 (2361)	19,894 (2361)	8.39 (1.19)
Draining Lymph Node	12.1 (6.5)	15	1,870 (184)	1,877 (184)	0.792 (0.099)
Non-draining Lymph Node	16.1 (14.2)	15	2,325 (218)	2,329 (218)	0.983 (0.109)
Esophagus	115 (102)	5	7,762 (1280)	7,764 (1280)	3.27 (0.60)
Spinal Cord	1.01 (0.09)	15	145 (22)	147 (22)	0.0619 (0.0129)
Thymus	9.63 (8.26)	5	1,545 (134)	1,546 (134)	0.652 (0.076)
Bone Marrow	65.8 (38.7)	30	7,988 (1,003)	7,991 (1,003)	4.60 (0.631)

Error is presented as SD for C_max_ and SEM for AUC.

a. AUC are from 0-1440 min except brain (0-960) and bone marrow (0-360) and partition coefficients with a corresponding plasma AUC

b. All infinity extrapolated AUC portions are <1%

## Abbreviations

ATR: Ataxia telangiectasia and Rad3-related
ATRi: Ataxia telangiectasia and Rad3-related inhibitor
XRT: Radiation Therapy
IR: Ionizing radiation
LC-MS/MS: Liquid chromatography-tandem mass spectrometry
PK: Pharmacokinetics
NCA: Noncompartmental Analysis
IS: Internal Standard
MRM: Multiple-reaction monitoring
IV: Intravenous
PO: Oral
AUC: Area Under the Curve
C_max_: Maximum plasma concentration
T_max_: Time of maximum plasma concentration
F: Bioavailability
