# Supplementary material for: Dose-Dependent Bioavailability and Tissue Distribution of the ATR Inhibitor AZD6738 (ceralasertib) in Mice
Source: Cancer Chemother Pharmacol. Author manuscript; Available in PMC 2023 Feb 1. (PMC8829872; doi:10.1007/s00280-021-04388-x)
Supplement: 1774452_Equation [file NIHMS1774452-supplement-1774452_Equation.pdf]

EQUATIONS

$$P_{Tissue} = \frac{Tissue\ AUC_{0-360}}{Parent\ AUC_{0-360}} \tag{1}$$

$$\%Excreted = \frac{A_{excreted}}{Dose} \tag{2}$$

$$Cl_R = \frac{A_{excreted}}{Plasma\ AUC_{0-360}} \tag{3}$$

$$MR = \frac{Metabolite\ AUC_{0-360}}{Parent\ AUC_{0-360}} \tag{4}$$

Predicted Bioavailability

$$F_H = 1 - E_H \tag{5}$$

$$E_H = \frac{(1 - fe) \cdot CL_{Blood}}{Q_{Liver}} \tag{6}$$

$$fe = \frac{Ae}{Dose} \tag{7}$$

$$CL_{Blood} = CL_{Plasma} \cdot \frac{AUC_{Plasma}}{AUC_{Blood}} \tag{8}$$

$$AUC_{Blood} = (1 - HCT) \cdot AUC_{Plasma} + HCT \cdot AUC_{RBC} \tag{9}$$

$$F_{Observed} = F_A \cdot F_G \cdot F_H \tag{10}$$

$$F_G = \frac{F_{Observed}}{F_H} \tag{11}$$

Non-Linear Compartmental PK Model with Saturable Absorption

$$\frac{dx_1(t)}{dt} = K_a x_4 - \frac{Cl}{V_C} x_1 - \frac{Cl_p}{V_C} x_2 + \frac{Cl_p}{V_P} x_2 \tag{12}$$

$$\frac{dx_2(t)}{dt} = \frac{Cl_p}{V_p} x_1 - \frac{Cl_p}{V_p} x_2 \tag{13}$$

$$\frac{dx_3(t)}{dt} = -K_a x_3 - K_{na} x_3 + \frac{V_{max}}{(K_m + x_4)} x_4 \tag{14}$$

$$\frac{dx_4(t)}{dt} = -K_a x_4 + K_a x_3 - \frac{V_{max}}{(K_m + x_4)} x_4 \tag{15}$$

Non-Linear Compartmental PK Model with Saturable Absorption with Uncouple Tumor Compartment

$$\frac{dx_1(t)}{dt} = K_a x_4 - \frac{Cl}{V_C} x_1 - \frac{Cl_p}{V_C} x_2 + \frac{Cl_p}{V_P} x_2 \quad (16)$$

$$\frac{dx_2(t)}{dt} = \frac{Cl_p}{V_p} x_1 - \frac{Cl_p}{V_p} x_2 \quad (17)$$

$$\frac{dx_3(t)}{dt} = -K_a x_3 - K_{na} x_3 + \frac{T_{max}}{(K_t + x_4)} x_4 \quad (18)$$

$$\frac{dx_4(t)}{dt} = -K_a x_4 + K_a x_3 - \frac{T_{max}}{(K_t + x_4)} x_4 \quad (19)$$

$$\frac{dx_5(t)}{dt} = k_{ct} x_1 - k_{ct} x_5 \quad (20)$$

SUPPLEMENTARY TABLES

Suppl.Table 1. Assay performance data of the calibration and QC samples for AZD6738 in Balb/c mouse plasma.

	Conc. (ng/mL)	Accuracy (%)	CV% (%)
Calibrators	0.3	99.4	5.7
	1	99.9	4.5
	3	101.4	3.6
	10	112.7	2.2
	30	104.8	1.8
	100	114.3	6.1
	300	95.9	1.4
	1000	98.0	4.2
QCs	0.8	91.2	4.2
	20	93.5	2.6
	800	89.1	2.9

From a single run, n=3 for each calibrator concentration and n=6 for each QC concentration.

Suppl.Table 2. Tissue and RBC AUC_{0-360} (μg/mL•min) from dose linearity and bioavailability studies.

Route	IV	PO	PO	PO	PO
Dose (mg/kg)	10	2.0	7.5	20	75
RBC	420 (44)	17.6 (2.4)	117 (17)	619 (72)	3,472 (433)
Liver	1,776 (93)	295 (25)	1,235 (100)	4,195 (570)	11,002 (578)
Kidney	905 (88)	87.7 (13.2)	421 (61)	1,364 (169)	4,911 (388)
Lung	332 (28)	51.6 (7.7)	320 (50)	1,066 (137)	4,642 (972)
Sk. Muscle	262 (31)	31.0 (2.7)	133 (6)	589 (93)	2,635 (445)
Brain	21.5 (1.8)	2.13 (0.37)	8.96 (1.03)	31.1 (5.8)	135 (18)

Error is reported as SEM

Suppl.Table 3. Tissue and RBC partitioning coefficient estimates from dose linearity and bioavailability studies.

Route<br>Dose (mg/kg)	IV<br>10	PO<br>2.0	PO<br>7.5	PO<br>20	PO<br>75	p[illegible]
RBC	1.28 (0.15)	0.771 (0.153)	0.905 (0.180)	1.14 (0.21)	1.55 (0.21)	0[illegible]
Liver	5.39 (0.39)	12.9 (2.1)	9.45 (1.49)	7.70 (1.55)	4.91 (0.39)	0[illegible]
Kidney	2.75 (0.30)	3.85 (0.80)	3.23 (0.64)	2.51 (0.48)	2.19 (0.22)	[illegible]
Lung	1.01 (0.10)	2.26 (0.47)	2.46 (0.51)	1.96 (0.38)	2.07 (0.45)	[illegible]
Sk. Muscle	0.794 (0.102)	1.36 (0.226)	1.02 (0.145)	1.08 (0.234)	1.18 (0.211)	[illegible]
Brain	0.0652 (0.0063)	0.0934 (0.0209)	0.0688 (0.0122)	0.0570 (0.0135)	0.0601 (0.0086)	[illegible]

All partition coefficients were calculated using $AUC_{0\text{-}360}$. Error is presented as SEM. Statistics were only calculated for PO treated groups.

NS=not significant

Suppl.Table 4. Physiological and PK parameters used for calculation of predicted contribution of liver to total bioavailability.

Parameter	Value
Q_{Liver} (mL/min) [a]	1.65
HCT[b]	0.554
Dose (µg)[c]	214
AUC_{Plasma} (µg/mL•min)	330
AUC_{RBC} (µg/mL•min)	421
AUC_{Blood} (µg/mL•min)	0.867
Cl_{Plasma} (mL/min)	0.634
Cl_{Blood} (mL/min)	0.550
Fraction excreted, *Fe*	0.0191
Amount excreted, *Ae* (µg)	4.00
Extraction ratio, E	0.337
Predicted bioavailability, $F_{Predicted}$	0.663

a. 90 mL/min/kg liver blood flow and a study based average mouse size of 18.3 g [50]
b. Per Charles River animal technical documentation
c. Average total dose administered IV based on an average mouse size of 18.3 g

Suppl.Table 5. Contribution of gut metabolism to oral bioavailability based on observed bioavailability and predicted hepatic contribution.

Dose (mg/kg)	$F_{Observed}$	F_{Liver}	F_{Gut}
2	0.457	0.663	0.689
7.5	0.588	0.663	0.887
20	0.855	0.663	1.29
75	0.952	0.663	1.44

Suppl.Table 6. Compartmental PK and model performance of AZD6738 non-linear model from dose linearity and bioavailability studies

Parameter	Estimate	CV%
Cl_t (mL/min/kg)	0.0366	7.6
V_c (L/kg)	1.91	17.0
Cl_p (mL/min/kg)	0.0269	22.8
V_p (L/kg)	2.61	13.0
K_a (min^{-1})	0.234	17.6
K_{na} (min^{-1})	0.0120	26.9
V_{max} (ng/min)	1238	31.5
K_m (ng)	157	32.8
SD slope	0.454	9.3
SD intercept [a]	0.0001	-
AIC [b]	1128	-
R^2 IV10	0.979	-
R^2 PO2	0.678	-
R^2 PO7.5	0.809	-
R^2 PO20	0.752	-
R^2 PO75	0.648	-

a. Intercept approximates 0 and was fixed to accommodate model
b. AIC: Akaike information criterion

Suppl.Table 7 Compartmental and NCA derived bioavailabilities

Dose (mg/kg)	Model F	NCA F
2	0.404	0.457
7.5	0.615	0.588
20	0.804	0.855
75	0.908	0.952

Data based on $AUC_{0-\infty}$ and actual dose. Bioavailability was calculated by pooling non-absorbed drug into a collection compartment and dividing it by the administered dose with results similar to those identified through NCA

Suppl.Table 8. Extensive Tissue Distribution Sulfoxide Metabolite Semi-Quantitative NCA

Route	PO
Dose (mg/kg)	75
C_{max}	6.46 (0.53)
C_{max}/dose	0.0730 (0.0060)
T_{max} (min)	120
AUC_{0-1440}	3,275 (572)
AUC_{0-1440}/dose	37.0 (6.5)
MR AUC_{0-1440}	1.38 (0.26)
Half-life (min)	129.7

Error is presented as SD for C_{max} and SEM for AUC.

Suppl.Table 9. Performance of compartmental model to fit plasma data from extensive PK study with uncoupled tumor compartment.

Parameter	Estimate	CV
Cl_t (mL/min/kg)	0.0416	8.1
V_c (L/kg)	5.04	14.6
K_a (min^{-1})	0.234	-
K_{tc} (min^{-1})	0.0537	36.3
K_{ct} (min^{-1})	0.325	40.5
SD slope, plasma	0.528	16.5
SD intercept[a], plasma	0.001	-
SD slope, tumor	0.496	16.6
SD intercept[a], tumor	0.0001	-
AIC [b]	882	
R^2 Plasma	0.883	
R^2 Tumor	0.705	

Ka, Clp, Vp, Kna, Vmax, and Km were all fixed to estimates from those in the dose linearity model.

a. Intercept approximates 0 and was fixed to accommodate model
b. AIC: Akaike information criterion

Suppl.Table 10. AZD6738 Balb/c mouse plasma protein binding.

Conc. (µg/mL)	Fu	CV%
1.00	0.345	4.94
10.0	0.311	1.16

N=3

LEGENDS TO SUPPLEMENTARY FIGURES

Suppl.Figure 1. Molecular structures of A) AZD6738 B) sulfoxide metabolite C) sulfone metabolite D) [$^{2}H_4$]-AZD6738 and proposed metabolic pathway [22].

Suppl.Figure 2. Analyte chromatograms from cross-validated LC-MS/MS assay. The bottom and middle traces are of the AZD6738 MRM channel in blank plasma (0 count offset) and 0.3 ng/mL LLQ (+1000 count offset), respectively, and correspond with the left y-axis. The top trace is [$^{2}H_4$]-AZD6738 MRM (+50,000 offset), corresponding to the right y-axis.

Suppl.Figure 3. Mean plasma and tissue concentration versus time profiles from bioavailability and dose linearity studies A) 10IV B) 2PO C) 7.5PO D) 20PO E) 75PO. Plasma (●), RBC (□), liver (△), kidney (◇), lung (○), brain (⊗). Error bars represent ± SD.

Suppl.Figure 4. Tissue partition coefficients from dose linearity and bioavailability studies A) RBC (p=0.0293) B) liver (p=0.0038) C) kidney (p=ns) D) lung (p=ns) E) skeletal muscle (p=ns) F) brain (p=ns). Partition coefficients were based on AUC0-360. Error bars represent ± SEM.

Suppl.Figure 5. Sulfoxide and sulfone metabolite identification in plasma and urine A) Example chromatogram 15 min plasma from a mouse in the 75PO group with AZD6738 shown as the bottom trace (+0 offset) and corresponding with the left y-axis and the sulfoxide metabolite as the top trace (+10,000 offset) corresponding to the right y-axis. Omitted is the MRM channel trace for the sulfone metabolite which produced no observable able peaks in plasma B) Example chromatograms of the sulfone MRM from the pooled urine of the 7.5PO group (bottom trace, no offset) and a vehicle treated group (top trace, +5,000 offset).

Suppl.Figure 6. Compartmental PK model performance showing observed concentration-time profiles observed plasma concentrations (○) and line displaying model prediction for A) 10IV B) 2PO C) 7.5PO D) 20PO E) 75PO and standardized residuals plotted as a function of time for F) 10IV G) 2PO H) 7.5PO I) 20PO J) 75PO.

Suppl.Figure 7. Concentration-time profiles from the extensive PK study for A) plasma B) RBC C) liver D) kidney E) spleen F) lung G) heart H) fat I) skeletal muscle J) brain K) tumor L) small intestine M) draining lymph node N) non-draining lymph node O) esophagus P) spinal cord Q) thymus R) bone marrow. Error bars represent ±SD.

Suppl.Figure 8. Extensive PK study A) AUC and B) partition coefficients. Error bars represent ± SEM.

Suppl.Figure 9. Mean plasma AZD6738 (○, µg/mL) and sulfoxide (□, IS normalized response) from extensive PK study.

Suppl.Figure 10. Non-linear compartmental model with uncouple tumor compartment A) model structure B) concentration-time profile for plasma with observed values (○) and line displaying model prediction C) concentration-time profile for tumor with observed values (○) and line displaying model prediction D) plasma standardized residuals plotted as a function of time E) tumor standardized residuals plotted as a function of time. Error bars represent ± SD.

SUPPLEMENTARY FIGURES

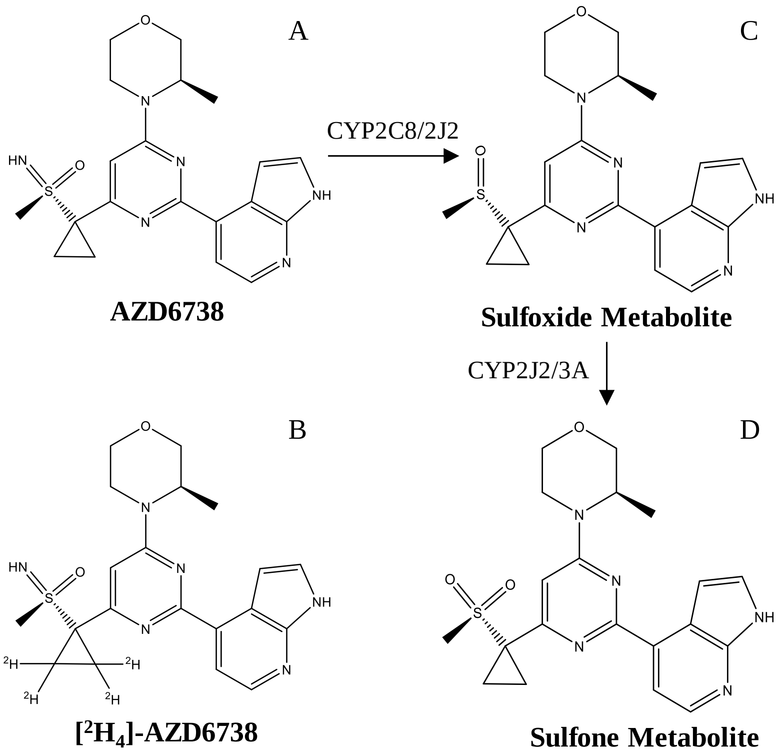

Suppl.Figure 1. Molecular structures of A) AZD6738 B) sulfoxide metabolite C) sulfone metabolite D) [$^2H_4$]-AZD6738 and proposed metabolic pathway [22].

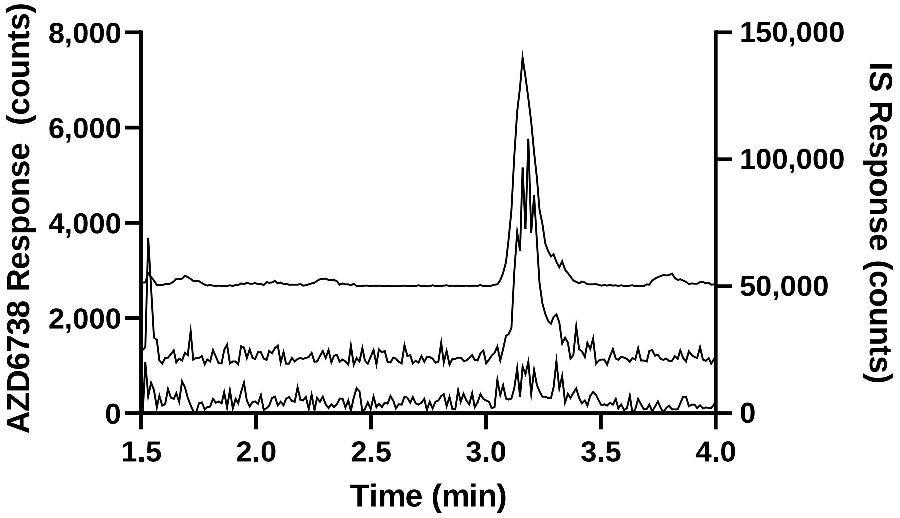

Suppl.Figure 2. Analyte chromatograms from cross-validated LC-MS/MS assay. The bottom and middle traces are of the AZD6738 MRM channel in blank plasma (0 count offset) and 0.3 ng/mL LLQ (+1000 count offset), respectively, and correspond with the left y-axis. The top trace is [$^{2}H_4$]-AZD6738 MRM (+50,000 offset), corresponding to the right y-axis.

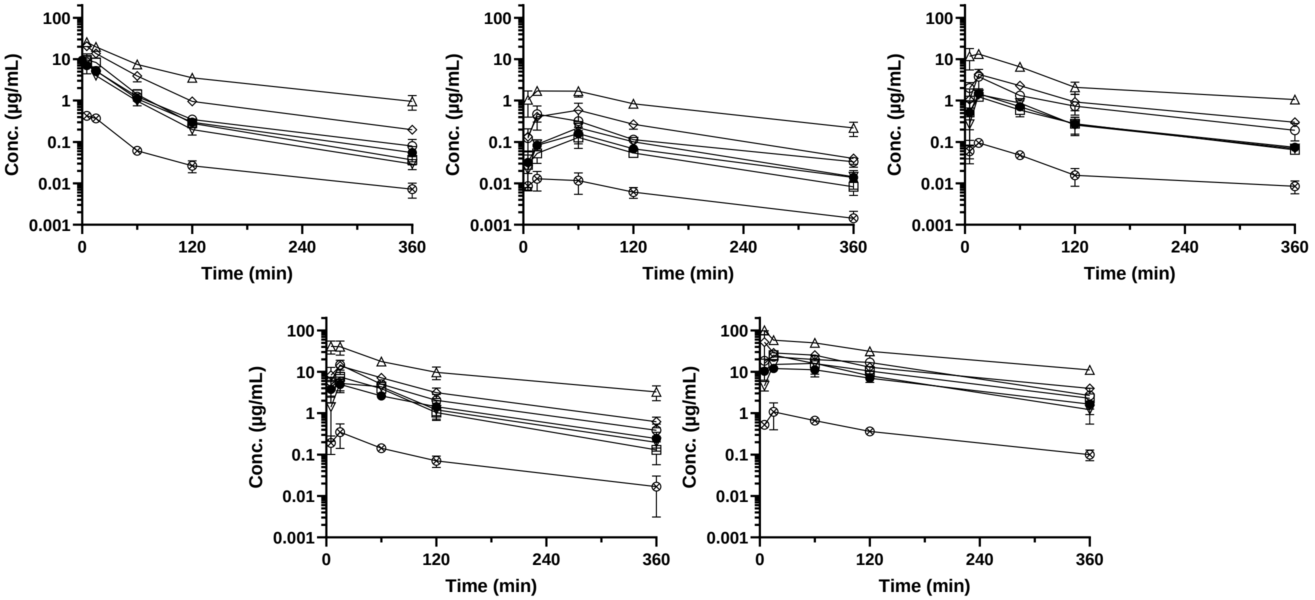

Suppl.Figure 3. Mean plasma and tissue concentration versus time profiles from bioavailability and dose linearity studies A) 10IV B) 2PO C) 7.5PO D) 20PO E) 75PO. Plasma (●), RBC (□), liver (△), kidney (◇), lung (○), brain (⊗). Error bars represent ± SD.

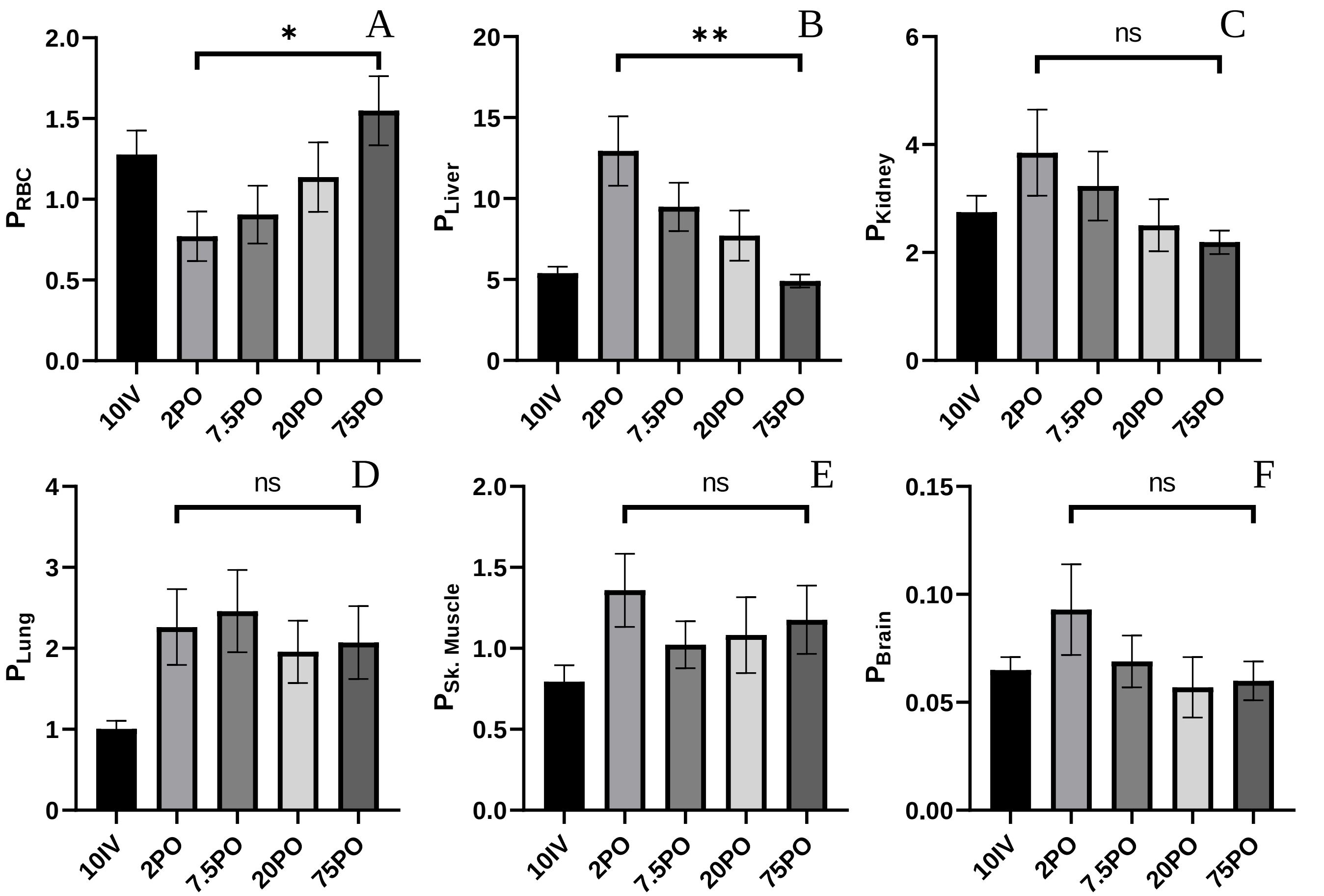

Suppl.Figure 4. Tissue partition coefficients from dose linearity and bioavailability studies A) RBC (p=0.0293) B) liver (p=0.0038) C) kidney (p=ns) D) lung (p=ns) E) skeletal muscle (p=ns) F) brain (p=ns). Partition coefficients were based on AUC0-360. Error bars represent ± SEM.

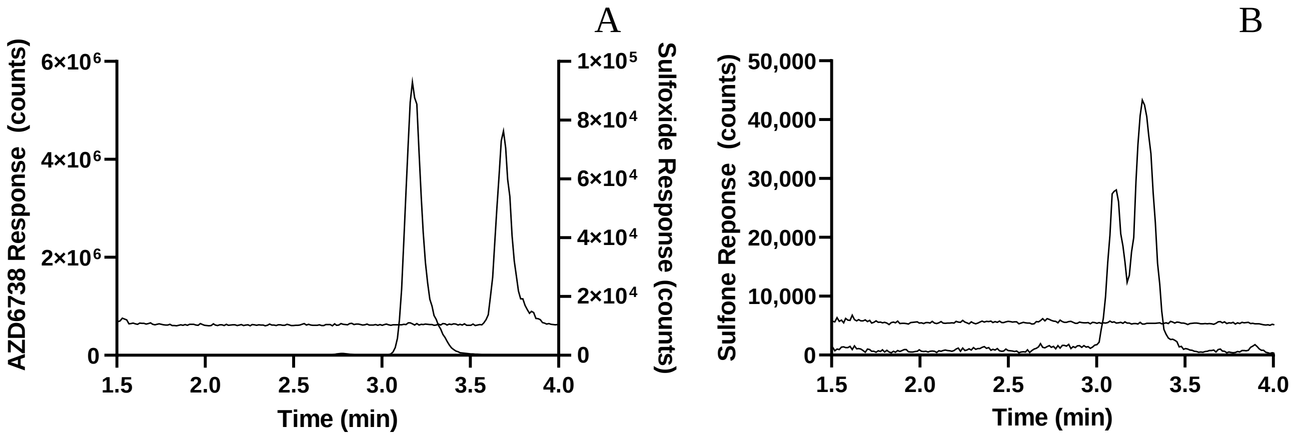

Suppl.Figure 5. Sulfoxide and sulfone metabolite identification in plasma and urine A) Example chromatogram 15 min plasma from a mouse in the 75PO group with AZD6738 shown as the bottom trace (+0 offset) and corresponding with the left y-axis and the sulfoxide metabolite as the top trace (+10,000 offset) corresponding to the right y-axis. Omitted is the MRM channel trace for the sulfone metabolite which produced no observable able peaks in plasma B) Example chromatograms of the sulfone MRM from the pooled urine of the 7.5PO group (bottom trace, no offset) and a vehicle treated group (top trace, +5,000 offset).

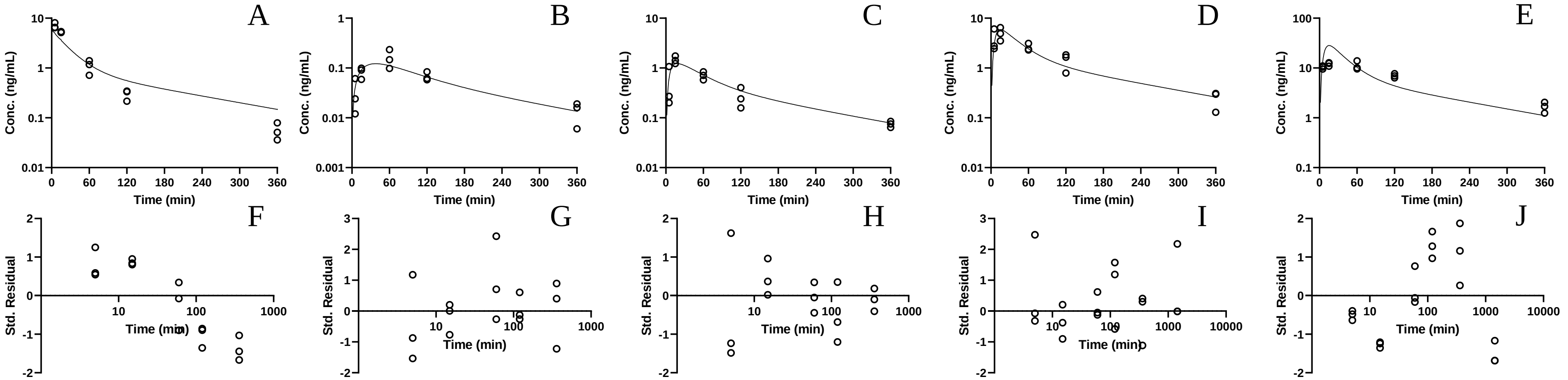

Suppl.Figure 6. Compartmental PK model performance showing observed concentration-time profiles observed plasma concentrations (◯) and line displaying model prediction for A) 10IV B) 2PO C) 7.5PO D) 20PO E) 75PO and standardized residuals plotted as a function of time for F) 10IV G) 2PO H) 7.5PO I) 20PO J) 75PO.

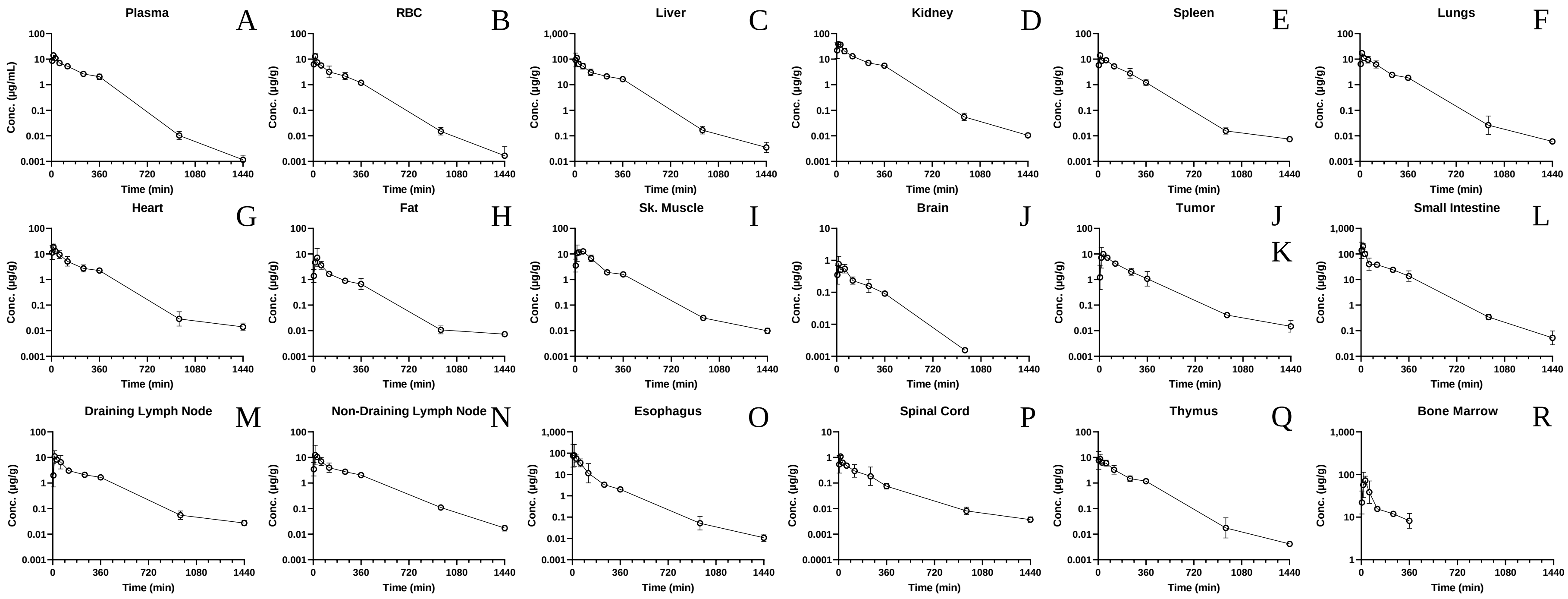

Suppl.Figure 7. Concentration-time profiles from the extensive PK study for A) plasma B) RBC C) liver D) kidney E) spleen F) lung G) heart H) fat I) skeletal muscle J) brain K) tumor L) small intestine M) draining lymph node N) non-draining lymph node O) esophagus P) spinal cord Q) thymus R) bone marrow. Error bars represent ±SD.

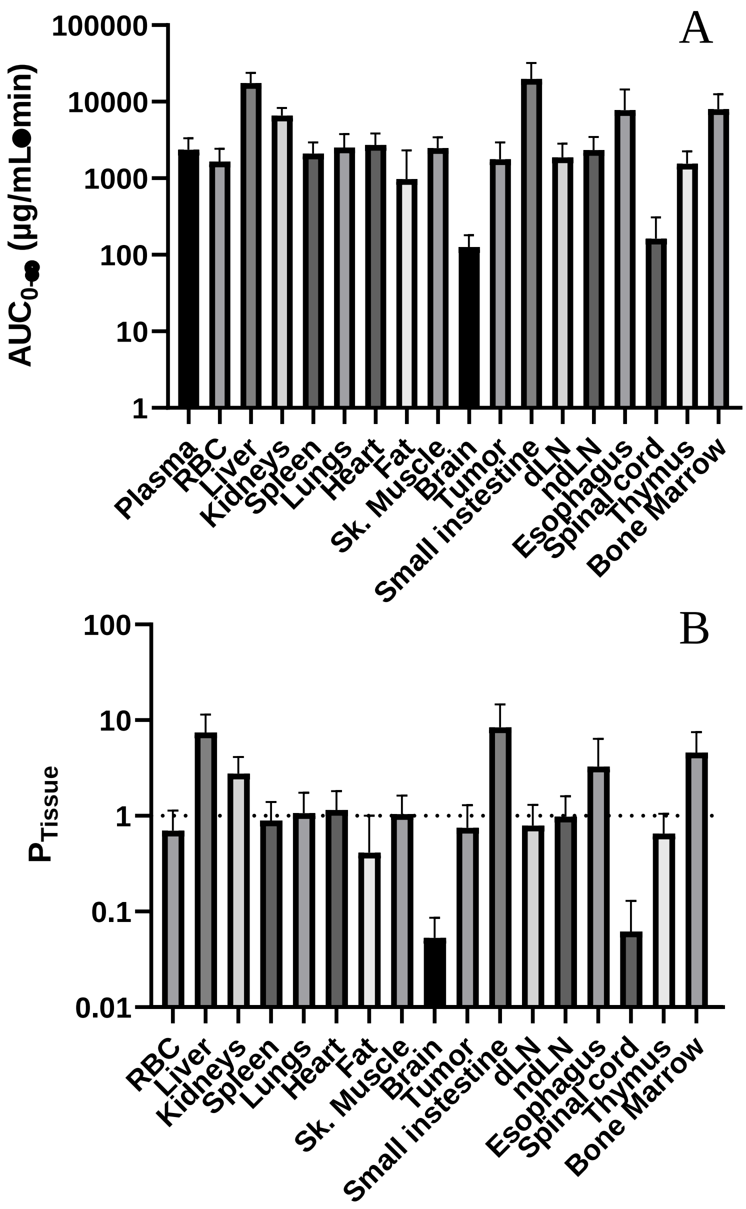

Suppl.Figure 8. Extensive PK study A) AUC and B) partition coefficients. Error bars represent ± SEM.

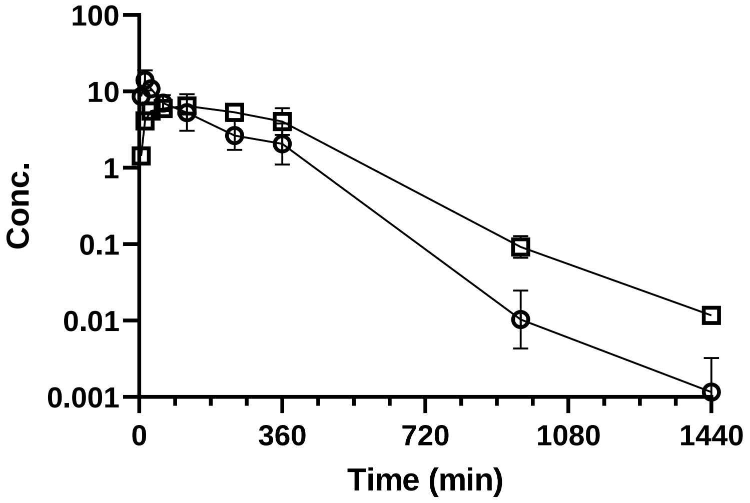

Suppl.Figure 9. Mean plasma AZD6738 (○, µg/mL) and sulfoxide (□, IS normalized response) from extensive PK study.

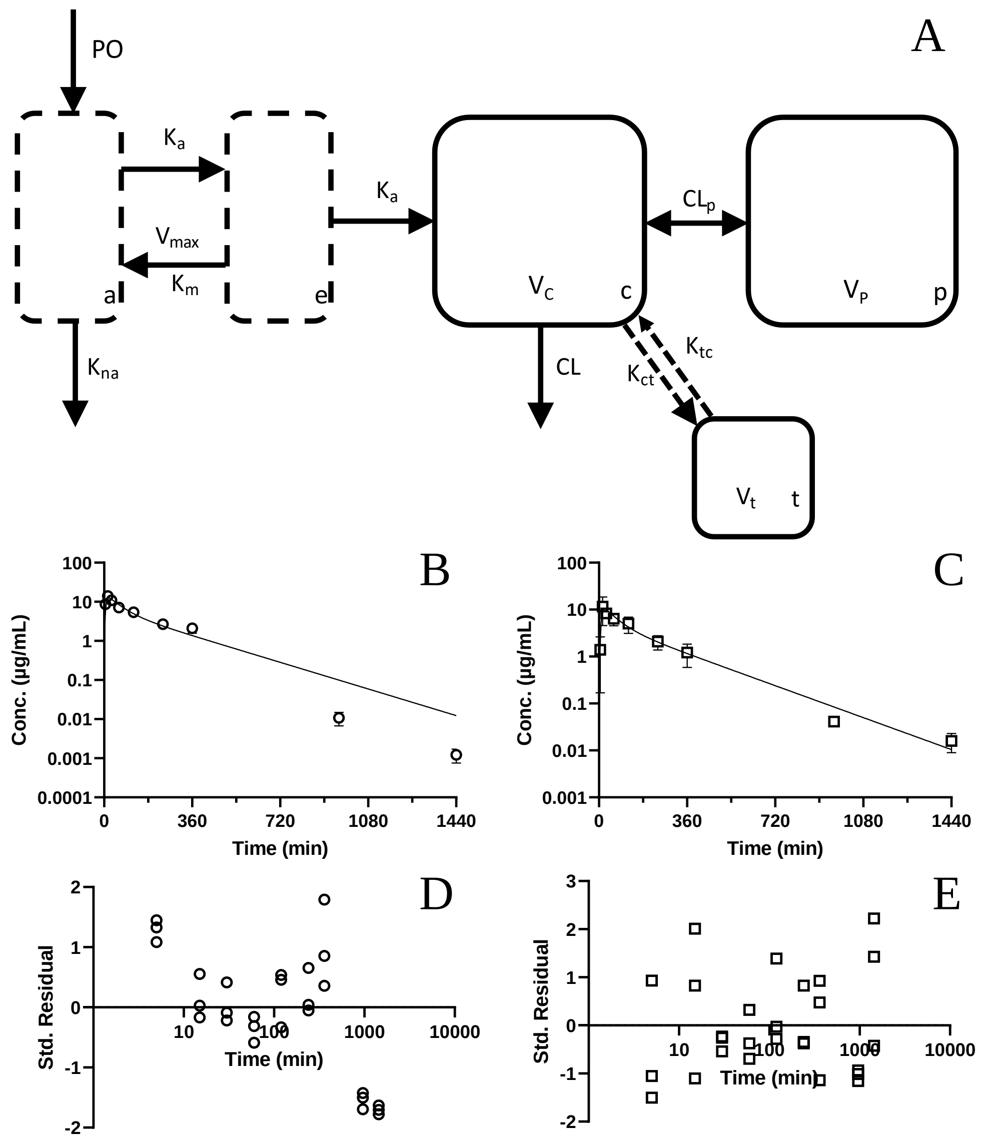

Suppl.Figure 10. Non-linear compartmental model with uncouple tumor compartment A) model structure B) concentration-time profile for plasma with observed values (○) and line displaying model prediction C) concentration-time profile for tumor with observed values (○) and line displaying model prediction D) plasma standardized residuals plotted as a function of time E) tumor standardized residuals plotted as a function of time. Error bars represent ± SD.
